# Targeting Chemokine Receptor CCR1 as a Potential Therapeutic Approach for Multiple Myeloma

**DOI:** 10.3389/fendo.2022.846310

**Published:** 2022-03-25

**Authors:** Annette Gilchrist, Stephanie L. Echeverria

**Affiliations:** ^1^ College of Pharmacy-Downers Grove, Department of Pharmaceutical Sciences, Midwestern University, Downers Grove, IL, United States; ^2^ Arizona College of Osteopathic Medicine, Midwestern University, Glendale, AZ, United States

**Keywords:** chemokine, CCR1, CCR1 antagonist, multiple myeloma, osteoblast, osteoclast, tumor microenvironment, bone

## Abstract

Multiple myeloma is an incurable plasma B-cell malignancy with 5-year survival rates approximately 10-30% lower than other hematologic cancers. Treatment options include combination chemotherapy followed by autologous stem cell transplantation. However, not all patients are eligible for autologous stem cell transplantation, and current pharmacological agents are limited in their ability to reduce tumor burden and extend multiple myeloma remission times. The “chemokine network” is comprised of chemokines and their cognate receptors, and is a critical component of the normal bone microenvironment as well as the tumor microenvironment of multiple myeloma. Antagonists targeting chemokine-receptor 1 (CCR1) may provide a novel approach for treating multiple myeloma. *In vitro* CCR1 antagonists display a high degree of specificity, and in some cases signaling bias. *In vivo* studies have shown they can reduce tumor burden, minimize osteolytic bone damage, deter metastasis, and limit disease progression in multiple myeloma models. While multiple CCR1 antagonists have entered the drug pipeline, none have entered clinical trials for treatment of multiple myeloma. This review will discuss whether current CCR1 antagonists are a viable treatment option for multiple myeloma, and studies aimed at identifying which CCR1 antagonist(s) are most appropriate for this disease.

## 1 Introduction

Multiple myeloma (MM) is the second most commonly diagnosed hematological malignancy in the world, and the incidence is rising (1). In the United States, between January 2010 and December 2016, the estimated prevalence of MM was 77,747 people with an incidence rate of 7.1 per 100,000 people (2). The 5-year survival rate of MM in all races for both sexes is 55.6% (3) more than double what it was in 1992 with the introduction of new targeted therapies and transplant techniques. Yet, the survival rate is still well below other hematologic cancers such as leukemia, non-Hodgkin’s lymphoma, and Hodgkin’s lymphoma. The etiology of MM is not fully understood and is likely multifactorial resulting from genetic predisposition and acquired mutations from insults such as ionizing radiation, organic chemicals, and toxins (4). Genetic abnormalities in oncogenes such as *CMYC*, *NRAS*, and *KRAS* may play a role in the development of plasma cell proliferation. MM displays aberrations similar to those seen in other cancers such as genomic instability, an altered metabolism, evasion of immune system surveillance, and drug resistance. These aberrations result in a disease that is difficult to treat without negative toxic effects to the patient.

Risk factors for MM include age, sex, and race. The median age of MM patients at diagnosis is 66-70 years (5). That MM occurs more often in older adults may be due to the accumulation of mutations requiring decades in the absence of other predisposing risk factors or mutations. MM occurs at a slightly higher frequency in men than in women and suggested underlying factors include discrepancies in health-risk behaviors, such as smoking, alcohol consumption, and obesity among men (6). The disease appears twice as frequently in African Americans as European Americans (7). This discrepancy is even higher among those below 50, indicating that African Americans have a younger onset of disease. Genome-wide association studies have identified several independent risk loci for MM (8) although no predisposing germline mutations have been found (9). There is evidence that systemic inflammation, oxidative stress, and exposure to radiation or environmental chemical agents may increase the risk of MM.

Patients afflicted with MM can experience symptoms of bone pain, back pain, abdominal pain, fatigue, nausea, vomiting, weakness, weight loss, thirst, recurrent infections, loss of appetite, headaches, and confusion (10). Evaluation for a MM diagnosis includes a complete blood count, complete metabolic panel, a urine and serum electrophoresis with immunofixation, quantification of M protein, a bone marrow examination using cytogenetic analysis or fluorescence *in situ* hybridization, and a skeletal survey for the identification of bone lesions (11). Initially, the International Staging System classified MM into three stages based primarily on serum β2 microglobulin levels and serum albumin levels (12). In 2015, the International Myeloma Working Group introduced a revised international staging system that classified patients into risk groups based on high-risk cytogenetic abnormalities and serum lactate dehydrogenase (LDH) levels (13). Treatment for MM is indicated once Calcium elevation, Renal impairment, Anemia, and Bone involvement, also known as the CRAB criteria, is met. Prognosis is dependent on the stage and biology of the disease and the survival rate has improved significantly in recent years (14).

The primary goal after diagnosis of MM is to achieve and maintain a complete response, meaning no M protein is present in the serum or urine. Current pharmaceuticals treat MM by targeting specific components of the bone and tumor microenvironment. The treatment for standard-risk MM is an initial combination chemotherapy of bortezomib, lenalidomide, and dexamethasone followed by autologous stem cell transplantation (ASCT) (10). For patients who are not candidates for ASCT due to age or other medical conditions a three drug combination (bortezomib, lenalidomide, and dexamethasone or the alternative regimen of daratumumab, lenalidomide, and dexamethasone) or two drug regimen (lenalidomide and dexamethasone) may be used (10, 15). Drugs currently used for the treatment of MM (**Table 1**) include proteasome inhibitors, immunomodulatory drugs (IMIDs), monoclonal antibodies (mAbs), histone deacetylase inhibitors (iHDACs), and nuclear export inhibitors. However, several of these drugs are limited in their capacity to treat MM due to toxicity issues, an inability to reduce tumor burden, and/or an inadequate increase of remission and/or survival times.

**Table 1:** Pharmaceutical classification, drug name examples, function in multiple myeloma, and limitations.

Drug Classification	Drug Names	Function in MM	Limitation	References
Alkylating Agents	Melphalan	Targeting MM cells, intercalating DNA, and inducing MM cell apoptosis	Grade 3 and Grade 4 adverse events	(16)
Proteasome Inhibitors (PI)	Bortezomib Carfilzomib Ixazomib	Promotion of MM apoptosis by suppression of NFκB signaling pathway, upregulation of NOXA, binding irreversibly to proteasome	High rates of discontinuation due to toxicity	(17)
Immunomodulatory Drugs (IMIDs)	Thalidomide Lenalidomide Pomalidomide	Enhancement of immune surveillance, downregulation of inflammatory environment, decreased MM growth, increased MM apoptosis	Poorly tolerated due to increased toxicity and secondary malignancies	(17)
Monoclonal Antibodies (mAbs)	Daratumumab Elotuzumab Isatuximab	Induction of MM cell apoptosis by binding to CD38 or SLAMF7 present on the MM cell surface	Infusion related reactions in 50% of patients	(18–21)
Histone Deacetylase Inhibitors (iHDACs)	Panobinostat Vorinostat Belinostat Romidepsin	Opening of the chromatin structure in MM cells, reactivation of the p21 tumor suppressor gene, and increased caspase mediated toxicity	Not viable for monotherapy as it cannot reduce tumor burden	(22, 23)
Nuclear Export Inhibitors	Selinexor	Inhibition of XPO1 from exporting tumor suppressor genes with suppression of NFκB and reduction of oncoprotein mRNA translation	Grade 3 and Grade 4 adverse events	(24)

Although recent advances have improved MM patient outcomes, successful treatment remains a challenge due to the tendency for patients to relapse and eventually become refractory to therapies (25). Thus, novel MM treatments are being actively pursued. One such pool for new targets for MM patients is the “chemokine network”, a collection of chemokines and chemokine receptors present in bone and MM tumor microenvironment. Chemokines are a group of small proteins (8-12 kDa), best known for their ability to provide directional guidance to migrating cells. They also play a role in neural regeneration, angiogenesis, cell activation, proliferation and differentiation, and cancer metastasis (26, 27). Chemokine-receptor 1 (CCR1) plays an extensive role in the bone microenvironment. Researchers have identified CCL3, an endogenous ligand of CCR1, as an osteoclast activating factor produced by MM plasma cells. CCL3 levels are elevated in myeloma patients and not only correlate with the extent of bone disease but are also inversely correlated to patient survival (28). Neutralizing antibodies to CCR1 inhibit CCL3 induced osteoclast formation in a dose-dependent manner and alter MM disease progression (29, 30). Furthermore, several CCR1 antagonists have been shown to reduce MM tumor burden in animal models (31–33). This review will discuss components of the chemokine network in the MM microenvironment and highlight recent studies of CCR1 antagonists.

## 2 Pathogenesis of Multiple Myeloma

### 2.1 Disease Classification

Originating in the bone marrow, plasma B cells are a specialized type of lymphocyte that produce antibodies, or immunoglobulins (Ig), in response to a pathogen. A typical Ig molecule consists of two heavy chains of one isotype (IgG, IgA, IgM, IgD, or IgE) and two light chains of lambda (λ) or kappa (κ). The heavy chains and light chains are bound together by disulfide bounds and if normal, are termed polyclonal proteins, as they arise from different clones of plasma B cells. MM is a malignant proliferation of plasma B cells that overproduce abnormal and defective Ig fragments known as monoclonal proteins (M proteins), as they are derived from the same clone. Normal plasma B cells develop from hematopoietic stem cells and undergo differentiation by V(D)J rearrangement in the bone marrow to result in a vast Ig collection. These cells then migrate to secondary lymphoid organs of the spleen or the lymph node to undergo affinity maturation, somatic hypermutation, and class-switch recombination to produce antibodies with high affinity for specific antigens.

MM begins in many patients as a monoclonal gammopathy of uncertain significance (MGUS), transitions to smoldering myeloma (asymptomatic MM), and becomes symptomatic multiple myeloma. MGUS is defined by the following three criteria: serum M protein (IgA, IgG, or IgM) <3 g/dL, clonal bone marrow plasma cells <10%, and absence of the CRAB criteria of hypercalcemia, renal insufficiency, anemia, and osteolytic lesions. MGUS can develop into smoldering myeloma defined as: M protein >3 g/dL and/or 10-60% bone marrow plasma cells, no amyloidosis, and no end-organ damage or other myeloma defining events. The final diagnosis of MM is the fulfillment of the following criterion: clonal bone marrow plasma cells ≥10% or biopsy proven bony or soft tissue plasmacytoma, and the presence of related organ or tissue impairment *via* CRAB criteria or presence of a biomarker associated with near inevitable progression to end-organ damage (34). In 2015, the International Staging System (ISS) was revised by the International Myeloma Working Group to better stratify patients with MM by including chromosomal abnormalities and serum LDH in order to provide improved prognostic value. The revised ISS (R-ISS) criteria for Stage I is a serum β2-microglobulin <3.5mg/L, serum albumin ≥3.5 g/dL, standard risk chromosomal abnormalities (CA) and normal LDH, Stage II is not R-ISS Stage I or III, and Stage III is a serum β2-microglobulin >5.5 mg/L and either high risk CA or high LDH. The 5-year overall survival rate for R-ISS Stage I is 82%, R-ISS Stage II is 62% and, R-ISS Stage III is 40% (13).

MM patients may be further classified by subtype based upon whether the malignant plasma cells produce Ig heavy chains plus light chains, light chains only, or neither. The distribution of various subtypes is 52% IgG, 21% IgA, 16% κ or λ chain only (Bence Jones), 2% IgD, 2% biclonal, 0.5% IgM, and 6.5% non-secretory or oligo-secretory MM (34). Subtyping of MM is important to understand which test values to monitor to determine disease progression. MM may also be characterized as either hyperdiploid or non-hyperdiploid. Non-hyperdiploid patients expressing chromosomal translocations at 14q32, with early translocation of the Ig heavy gene (IgH), creates an increased expression of IgH and overall worse prognosis compared to hyperdiploid. Half of MM patients are hyperdiploid with extra copies of odd numbered chromosomes, including trisomies of the chromosomes 3, 5, 7, 9, 11, 15, 17, and 21 and is correlated with better survival rates than their non-hyperdiploid counterparts (35, 36). Both non-hyperdiploid and hyperdiploid result in the dysregulation of G_1_/S cell cycle and cyclin D gene transcription, a genetic alteration which contributes to the onset of human cancers *via* uncontrolled cell proliferation (37). A subset of MM patients are categorized as high-risk based on the presence of patient- and disease-based factors such as frailty, extramedullary disease, cytogenetic abnormalities, elevated LDH levels, renal impairment, or relapses occurring earlier than expected. These patients continue to have inferior outcomes despite the advances in MM treatment over the last decade (38).

### 2.2 Bone Marrow Microenvironment

A major feature of MM is osteolytic bone disease with approximately 60% of MM patients experiencing bone pain, primarily in the central skeleton, and 20-25% of MM patients effected by pathological fractures, compression factors, and osteoporosis (34). The bone microenvironment (BME) plays a crucial role in the establishment and progression of MM as bone diseases of osteopenia and lytic lesions are caused by the imbalance between bone regeneration and bone resorption (39). A stable BME is critical for the appropriate maintenance of normal cell proliferation, metabolism, differentiation, and mobilization (40). The BME is comprised of a cellular compartment, an extracellular matrix (ECM), and a non-cellular compartment or “liquid” compartment. The cellular compartment consists of hematopoietic and non-hematopoietic cells including bone marrow stromal cells (BMSCs), osteoblasts (OBs), osteoclasts (OCs), effector immune cells, endothelial cells, fibroblasts, and adipocytes. The extracellular matrix is a complex network of collagen, fibronectin, and laminin whilst the liquid compartment contains growth factors, cytokines, and chemokines (41).

Normally, there is a delicate balance in the number of OCs, OBs, and osteocytes to maintain bone homeostasis. The BME has growth factors such as receptor activator of nuclear factor kappa-B ligand (RANKL), interleukin 3 (IL-3), and macrophage inflammatory protein-1 alpha (MIP-1α; aka CCL3) that lead to osteoclastogenesis. While RANKL inhibits OC apoptosis, the soluble form of its receptor RANK has been shown to accelerate bone loss. The BME is also the primary source of interleukin 6 (IL-6), a cytokine associated with stimulating cytotoxic T-cells and important to differentiation of OC precursors to mature and active OCs (40). Other players attributed to perpetuating IL-6 production include interleukin 1β (IL-1β), tumor necrosis factor α (TNF-α), nuclear factor kappa-light-chain-enhancer of activated B cells (NF-κB), and the Notch signaling pathway (39). An insulin-like growth factor (IGF) system with six high-affinity IGF binding proteins (IGFBP1-6), IGF-1, IGF-2, and IGF-1R, is found in the ECM compartment (40). Notably, IGF-1 has been shown to play a key role in the enhanced proliferation and survival of MM cells.

### 2.3 Tumor Microenvironment

#### 2.3.1 General Features

**Figure 1:**
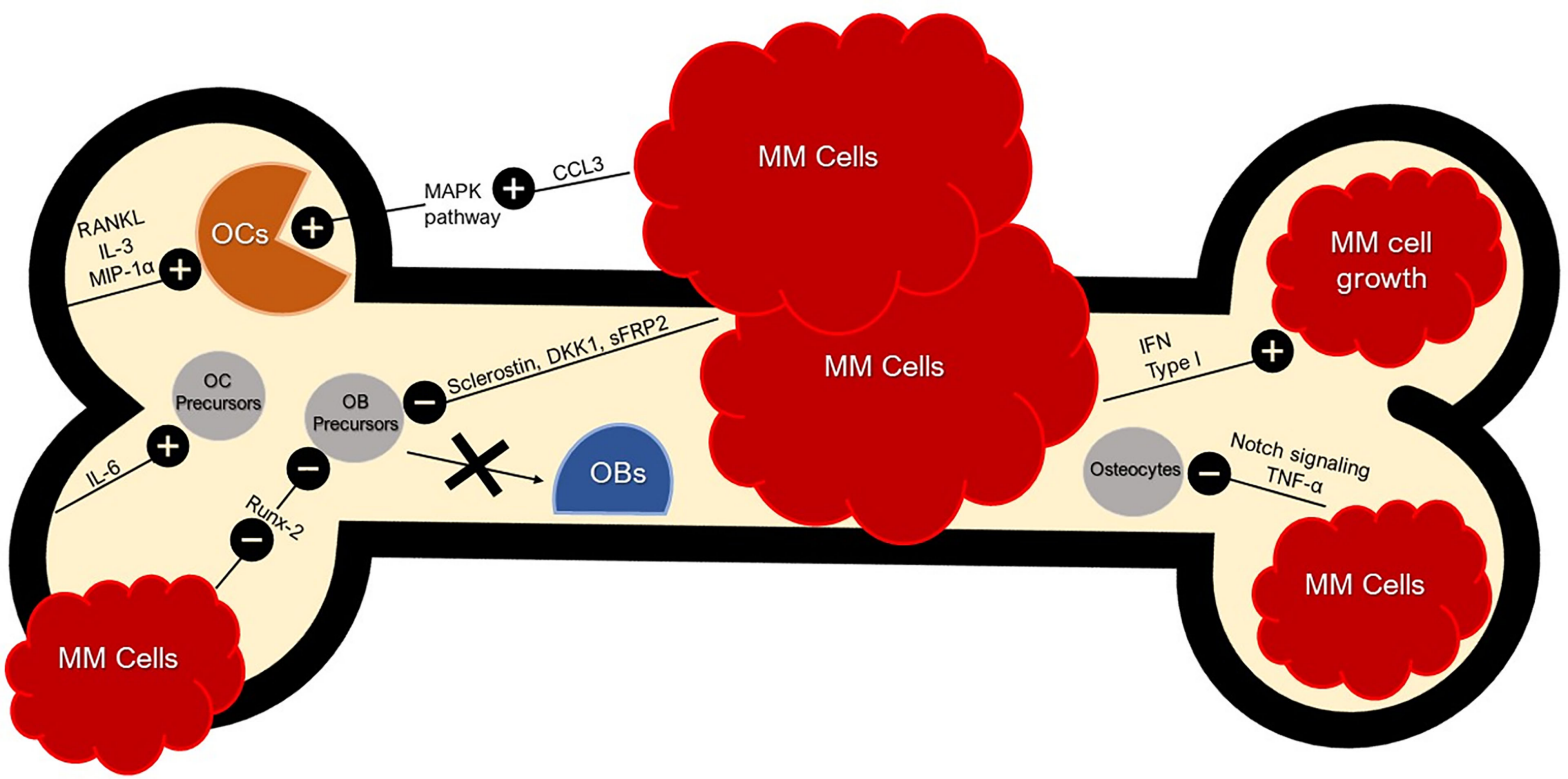
Bone and Tumor Microenvironment. Bone and Tumor Microenvironment. BME produces RANKL, IL-3, and MIP-1α (CCL3) for OC activation. RANKL leads to the inhibition of OC apoptosis. IFN Type 1 secreted by MM cells favors MM growth and immunosuppression. CCL3 secreted by MM cells activate the MAPK pathway, further stimulating osteoclastogenesis. MM cells can inhibit OB differentiation with sclerostin and DKK1 by dysregulating the Wnt signaling pathway; an essential pathway for osteoblastogenesis. MM cells also secrete sFRP-2 which suppresses OB differentiation. MM cells inhibit Runx-2 in OB precursors and thus inhibit OB maturation. MM cells inhibit osteocytes *via* abnormal apoptosis by Notch signaling which is sustained by TNF-α. Crosstalk between BMSCs and MM cells induce pro-osteoclastogenic factors such as IL-6. MM cell secretion of CCL3 binds to CCR1 and CCR5 on OCs, enhancing OC activity.

During initial disease development, malignant clonal plasma cells establish themselves in bone marrow niches that support their growth. In normal BME, natural killer (NK) cells and cytotoxic T lymphocytes (CTLs) are capable of targeting and attacking tumors to initiate the anti-tumor response, but in the MM microenvironment this response is diminished (42). Standard components of the BME can inadvertently prove conducive to the growth and adhesion of MM plasma cells. Additionally, MM may drive more optimal conditions by upregulating or downregulating certain components of the BME (**Figure 1**). For instance, MM plasma cells can secrete interferon (IFN) type I, a protein shown to promote immunosuppression and favor MM growth (43). MM plasma cells are able to adhere to vascular cell adhesion molecule-1 (VCAM-1) on BMSCs *via* integrins such as lymphocyte function-associated antigen-1 (LFA-1) and very late antigen-4 (VLA-4) that in turn can induce a favorable environment for the growth, proliferation, invasion, and drug resistance (44). BMSCs can produce Jagged, thereby activating the Notch pathway in MM plasma cells and contributing to MM cell proliferation, survival, migration, and bone disease (45). The adhesion of MM plasma cells to BMSCs can stimulate the secretion of factors such as B-cell activating factor (BAFF) from BMSCs that in turn upregulates the expression of anti-apoptotic proteins like myeloid cell leukemia-1 (MCL-1) and B-cell lymphoma-2 (BCL-2), and cell cycle regulating proteins like serine/threonine kinase Pim-2 in MM plasma cells that may lead to chemoresistance (39).

Both BMSCs and MM plasma cells can secrete vascular endothelial growth factor (VEGF), IGF-1, interleukin 1 (IL-1), transforming growth factor beta (TGF-β), angiopoietin-1 (Ang-1), platelet derived growth factor (PDGF), basic-fibroblast growth factor (bFGF), and hepatocyte growth factor (HGF) which have been shown to promote osteoclastogenesis, tumor growth, and angiogenesis in MM (39). VEGF is also able to stimulate chemotaxis of BMSCs *via* the VEGF-receptor-1 (VEGFR-1) with increased expression of VEGFR-1 being a common attribute in MM patients. VEGF may also interact with the pro-angiogenic factor osteopontin, secreted by OCs, to induce OC activity and enhance angiogenesis (40). The bone marrow also contains “sinusoids” which can support MM cells with an increased delivery of O_2_ and increased removal of catabolites due to the abundant blood vessels present nearby (40).

Myeloid derived suppressor cells (MDSCs) are a population of immature myeloid cells that normally differentiate into macrophages, granulocytes, and dendritic cells (DCs). In the MM environment, differentiation is inhibited and MDSCs accumulate. As MDSCs accumulate, they suppress T-cell proliferation through release of arginase, reactive oxygen species, and nitric oxide (42). DCs are bone marrow derived professional antigen presenting cells that assist T-cells in the immune response in a normal environment. However, in the MM microenvironment DCs are functionally impaired. In the MM microenvironment secretion of chemokines and cytokines can also recruit regulatory T-cells and promote immunosuppression (42).

The process of dissemination is a critical feature of disease progression in multiple myeloma. It is a multi-step process requiring release from the supportive bone marrow niche, intravasation into nearby blood vessels, and extravasation and homing to another bone marrow site. Most newly diagnosed MM patients have detectable circulating MM plasma cells with higher numbers being an independent predictor of shorter progression-free survival and overall survival (46). Notably, the dissemination of myeloma cells is a key feature of aggressive, advanced forms of MM, including extramedullary disease and plasma cell leukemia, both of which have a poorer prognosis.

#### 2.3.2 Osteoblasts, Osteocytes, and Osteoclasts

Osteolytic bone disease is the hallmark of MM. The interaction between MM plasma cells and the BME result in activation of OC and suppression of OB with subsequent bone loss. CCL3 secreted by MM plasma cells can activate the mitogen-activated protein kinase pathway (MAPK) and stimulate osteoclastogenesis (39). MM plasma cells inhibit OB differentiation through the secretion of CCL3, sclerostin and dickkopf1 which in turn dysregulates Wnt signaling, a signaling pathway essential for osteoblastogenesis. MM plasma cells may secrete the soluble Wnt inhibitor frizzled-related protein 2 (sFRP-2) which has shown to suppress OB differentiation in the majority of MM human cell lines including RPMI8226 and U266 (40). In addition, MM plasma cells may inhibit runt-related transcription factor-2 (Runx-2) in OB precursors and inhibit maturation.

In recent years, osteocytes have emerged as key regulators of bone loss in MM. MM patients have reduced numbers of osteocytes compared to healthy controls and this correlates with the extent of MM-induced disease. MM plasma cells inhibit osteocytes *via* abnormal apoptosis driven by Notch signaling with TNF-α sustaining this activation (47). Osteocyte apoptosis appears to be crucial in modifying the BME that favors MM plasma cell homing and growth (48).

The adhesion of MM plasma cells to bone marrow upregulates the production of important growth factors including IL-6, RANKL, activin-A, and macrophage colony stimulating factor (MCSF), which together are responsible for increased differentiation and maturation of OCs (40). The increased RANKL expression, once bound to its respective receptor, increases OC differentiation by the NF-κB pathway and c-Jun N-terminal kinase pathway (40). Adhesion of MM plasma cells to bone marrow also upregulates production of CCL3 which binds to CCR1 and CCR5 expressed by OCs and leads to enhanced OC activity (47).

#### 2.3.3 Dissemination

CXCR4 affects MM plasma cell mobilization and egression out of the bone marrow. When MM cells adhere to BMSCs, CXCL12 up regulates its own secretion, which further up regulates VEGF and IL-6 secretion and thus promotes enhanced homing through further expression of integrins. To enter into the circulation, MM plasma cells must overcome adhesive interactions that act as a bone marrow retention signal. Studies have shown that there are decreased levels of the activated form of integrin β1, Syndecan-1, and CD40 in MM plasma cells in the peripheral blood compared with those in the bone marrow of MM patients (46). Recently, Zeissig et al. demonstrated that CCR1 is a crucial driver of MM plasma cells dissemination *in vivo* (33). CCL3/CCR1 signaling may play a role in desensitizing MM plasma cells to CXCL12 thus facilitating their release from the bone marrow niche. As MM plasma cells exit the bone marrow they continue to secrete pro-osteoclastogenic cytokines and enhance bone resorption with subsequent release of growth factors that further perpetuate the growth of MM; this positive feedback loop is known as the “vicious cycle” (47).

#### 2.3.4 Genomic Instability

Genomic instability is a hallmark of cancer with four main types: chromosomal instability, intrachromosomal instability, microsatellite instability, and epigenetic instability. Yet, like the theory of the chicken and the egg, it is unclear as to whether cancer causes the genomic instability, or the genomic instability precedes the onset of cancer. However, it is clear that crosstalk between the tumor microenvironment and BME, leads to the differential gene expression required to support tumor proliferation (49). In MM, chromosomal instability, point mutations, and microsatellite instability are the most prominent genomic aberrations. Dysfunctional homologous recombination and the activation of CD40 and IL-4 by MM cells has shown to create DNA double-strand breaks to further exacerbate the genomic instability (49). MM patients also show an increased level of activin A which is not secreted by MM cells or normal BMSCs, suggesting there may be a genetic defect on malignant BMSCs causing the increased release of activin A, thereby increasing OC activation and inhibiting OB differentiation. BMSCs of MM patients also exhibit other abnormal gene expressions such as growth differentiation factor-15 which in turns supports MM cell survival and self-renewal (39).

#### 2.3.5 Altered Metabolism

Multiple changes in metabolism occur in MM. For example, MM plasma cells demonstrate abnormally high glucose intake, with subsequent enhanced glycolysis and lactate production. The final step of glycolysis involves the conversion of phosphoenolpyruvate into pyruvate and ATP, and this step is catalyzed by pyruvate kinase. A particular isoform of pyruvate kinase, termed PKM2, is upregulated in MM plasma cells. Silencing of PKM2 has been shown to decrease MM growth and results in cell cycle arrest at the G_1_/S transition (50). The transcription factor hypoxia-inducible factor-1 (HIF-1) is upregulated in MM plasma cells where it plays a role in the accumulation of increased glycolytic metabolites as it induces transcription of several genes that upregulate glycolytic enzymes and lactate production (51). Altered metabolism may also play a role in MM drug resistance as enhanced glucose metabolism is linked to drug resistance. For example, Maiso et al. demonstrated that inhibition of lactate dehydrogenase A and HIF1-a can restore drug sensitivity to anti-myeloma agents such as bortezomib (51).

MM plasma cells lack the ability to create their own glutamine, and are reliant on extracellular glutamine uptake (52). As a result, they consume huge amounts of glutamine and the amino acid concentration is lower than normal. Glutamine depletion hinders OB differentiation. Human MM cell lines are often “glutamine addicted” and vulnerable to cytotoxic effects once glutamine is depleted. The *MYC* oncogene is present in MM plasma cells and is involved in glutaminolysis by enhancing the expression of glutamine transporters (50).

That obesity is a risk factors for MM, and MM plasma cells are often found in an environment with relatively high adiposity, led some investigators to suggest that bone marrow adipocytes may enhance MM engraftment in the BME (53). Researchers have shown bone marrow adipocytes isolated from MM patients support myeloma growth and enhance chemoresistance *via* secretion of adipokines, such as leptin and adipsin (54). Elevated plasma levels of free fatty acids (FFAs) are thought to restrict glucose utilization and induce insulin resistance. Plasma FFA concentrations are primarily governed by lipolysis in adipocytes. MM plasma cells can induce lipolysis in adipocytes and the released FFAs are then taken up by MM plasma cells through fatty acid transporters 1 and 4 (55).

#### 2.3.6 Immune Evasion and Drug Resistance

Impaired immune surveillance, including increased numbers of immunosuppressive cells, is thought to be critical for MM disease progression. In addition, MM plasma cells are apoptosis resistant, which has led some investigators to look at the interactions between Fas and its ligand (FasL), components in the extrinsic apoptotic system. FasL, also known as CD178, is expressed on CTLs and functions by engaging the death receptor Fas (CD95) and triggering apoptosis. Alexandrakis and colleagues proposed that serum levels of soluble Fas-L (sFas-L) may reflect MM disease progression (56). However, FasL antibodies were unable to fully restore Fas in the MM and CTL environment, suggesting other factors play a part (57). The interaction between MM plasma cells and the BME can render myeloma cells resistant to current regimens of pharmacologic therapy, a mechanism coined, “cell adhesion-mediated drug resistance” (CAM-DR) (58). This interaction can also facilitate immune evasion to avoid CTL lysis by “cell adhesion-mediated immune resistance” (CAM-IR) (57). CAM-DR is crucial to induction of CAM-IR with the subsequent response being a dampening of the immune response through reduced adhesion molecule expression. CAM-IR can also induce a downregulation of Fas on CTLs. With the use of integrins, MM cells can adhere to fibronectin in the ECM as an effective evasion technique to avoid drug-induced apoptosis (41). Macrophages may also assist MM cells through contact-mediated and non-contact mediated interactions that result in the protection of MM cells (42). MM cell interaction with the BME is believed to play role in drug resistance and as a result, targeting this interaction may provide a promising therapeutic strategy. However, we don’t yet have a comprehensive understanding of which receptor-ligand systems and subsequent downstream signals are responsible for the resilience of MM cells.

## 3 Current Pharmacological Treatments

The treatment of MM is typically a two- or three- drug combination with historically one of the drugs being an alkylating agent such as melphalan. Melphalan functions by targeting MM cells, permanently intercalating their DNA, and inducing cell apoptosis (59). In oncology/hematology clinical trials, safety endpoints are defined by adverse events rated on a scale of Grade 1-5, with Grade 1 defined as requiring no intervention and Grade 5 resulting in death. As melphalan is commonly used in other drug combinations, adverse events of Grade 3 and Grade 4 consisting of neutropenia (neutrophils <1000/mm^3^ to 500/mm^3^ or <500/mm^3^), thrombocytopenia (platelets <50,000/mm^3^ to 25,000/mm^3^ or <25,000/mm^3^), anemia (hemoglobin < 8.0 g/dL; transfusion indicated or life threatening consequences), and pneumonia (severe but not life threatening or life threatening) are seen (18). Melphalan with prednisone was once the standard treatment but due to melphalan’s interference with adequate stem cell mobilization, triple drug combinations such as bortezomib, lenalidomide, and dexamethasone are currently preferred (44, 59).

Bortezomib, carfilzomib, and ixazomib, are proteasome inhibitors that promote apoptosis by suppression of the NFκB signaling pathway, upregulation of NOXA, a pro-apoptotic member of the BCL-2 protein family, or binding irreversibly to the proteasome which results in the upregulation of apoptosis in MM cell lines (59). Drug combinations with bortezomib display a high degree of efficacy but are associated with high rates of discontinuation due to toxicity (60). Thalidomide, lenalidomide, and pomalidomide are IMIDs that function by enhancing immune surveillance and changing the tumor microenvironment (59). IMIDs modulate the tumor microenvironment in part through downregulation of proinflammatory cytokines IL-6, IL-1β, TNF-α, IL-1, and IL-12 and increase of anti-inflammatory IL-10. IMIDs limit MM cell growth with the downregulation of bFGF and VEGF and increase T-cell proliferation with the upregulation of IFN-γ and IL-2 (59). However, the use of IMIDs can be poorly tolerated due to toxicity and secondary malignancies (61).

Daratumumab, isatuximab, and elotuzumab are mAbs engineered to enhance the immune system response. These mAbs induce cell death by binding to specific antigens such as CD38 (daratumumab, isatuximab) or SLAMF7 (elotuzumab) found on the surface of MM cells and inducing direct activation of NK cells, antibody dependent cell mediated toxicity, complementary dependent cytotoxicity, or antibody dependent cellular phagocytosis (59). Adverse events are considered manageable but approximately half of all patients treated with daratumumab report infusion-related reactions (19).

Panobinostat, vorinostat, belinostat, and romidepsin are iHDACs that function by opening the chromatin structure of MM cells, reactivating the previously silenced p21 tumor suppressor gene, and increasing caspase mediated toxicity (59). However, iHDACs alone do not induce tumor regression and cannot be used as a monotherapy for the treatment of MM (22).

Selinexor is a nuclear export inhibitor that inhibits XPO1 from exporting tumor suppressor proteins leading to suppression of NF-κB and the reduction of oncoprotein mRNA translation (59). Selinexor with dexamethasone is a potent treatment option for aggressive myeloma but is associated with Grade 3 and Grade 4 adverse events of thrombocytopenia, anemia, neutropenia, bleeding, and infection (24).

While advances in therapies have increased the 5 year survival rates for patients with standard-risk MM, they have shown more limited benefits for high-risk patients. For example, iMID/PI-refractory patients who have received at least three prior lines of therapy regimens and have been exposed to an alkylating agent have a median overall survival of only 13 months from the double-refractory state (62). Thus, effective treatments that target novel pathways with minimal toxicities are still needed.

## 4 Role of the Chemokine Network in Multiple Myeloma

### 4.1 Chemokines and Chemokine Receptors

Chemokines are defined by their primary amino acid structure sequence and the arrangement of cysteine residues to form an overall structure consisting of a central three stranded β-sheet, an overlying C-terminal α-helix, and a short unstructured N terminus that plays a key role in receptor activation (63). Based upon the spacing of conserved cysteine residues, chemokines are typically divided into four subfamilies, with two major subfamilies and two minor subfamilies. The major subfamilies consist of CC with two cysteines next to each other and CXC with two cysteines separated by one amino acid. The minor subfamilies consist of CX3C with two cysteines separated by three amino acids and XC with the first cysteine lacking. In humans, there are 27 CC chemokines, 17 CXC chemokines, 2 XC chemokines, and 1 CX3C chemokine, for a total of 47 different chemokines. All chemokines are soluble proteins with the exceptions of CXCL16 and CXCL1 which remain tethered to the cell surface (27).

Chemokines serve as ligands to the chemokine receptors which belong to the G-protein coupled receptor (GPCR) superfamily. The transmembrane, heptahelical proteins are primarily expressed on leukocytes but can also be found on several other cell types (26). Chemokine receptors are named based on the type of chemokine they bind. For example, receptors CCR1-CCR10 bind CC chemokines, receptors CXCR1-CXCR6 bind CXC chemokines, receptor XCR1 binds C chemokine, and receptor CX3CR1binds CX3C chemokine. Four atypical chemokine receptors (ACKR1 – ACKR4) lack the ability to engage traditional downstream signaling pathways and rather are thought to serve as chemokine scavengers (27). Whilst some chemokine-chemokine receptor interactions are highly specific such as CXCL16 which interacts only with CXCR6, many chemokines are “promiscuous” binding to multiple receptors, as is observed for CCL7 which can bind to CCR1, CCR2, CCR3, CCR5, ACKR1, and ACKR2. It was initially thought that the existence of multiple chemokines and chemokine receptors resulted in biochemical redundancy. However, many now argue that a large chemokine family is a sophisticated strategy to fine-tune the leukocytic response to different inflammatory stimuli (50).

Chemokine receptors can induce intracellular signaling by both G-protein dependent and G-protein independent pathways. In G-protein dependent signaling, as the chemokine(s) bind to the receptor(s), a GDP/GTP exchange on the Gα subunit occurs, causing a disassociation of heterotrimeric Gαβγ into an active GTP-bound Gα and Gβγ dimer that are each able to activate downstream signaling pathways such as the activation of Rac, Rho, and CDC42, and the inhibition of adenyl cyclase (64). Activation of chemokine receptors by chemokines may also recruit β-arrestin which in turn activates G-protein independent pathways such as Akt, p38, AMPK, and ERK 1/2 (65). CCR1 is a receptor that can phosphorylate ERK 1/2, protein kinases which contribute to the Ras-Raf-MEK-ERK MAP kinase signaling pathway, a pathway with a large role in cell apoptosis and proliferation. The MAP kinase cascade in particular, is considered to be the most important oncogenic driver of human cancers (66).

Chemokines, particularly in their oligomeric form, bind to glycosaminoglycans (GAGs) which are polysaccharides present on the surface of most cells. GAGs are divided into four groups based upon their repeating disaccharide units: heparin/heparan sulfate, chondroitin sulfate/dermatan sulfate, keratan sulfate, and hyaluronic acid (67). The interactions between GAGs and chemokines is believed to maintain high local concentrations of chemokines and establish concentration gradients that promote chemotaxis. Genetic variants in GAGs that result in defective GAG binding, lead to impaired cell migration. Some researchers have suggested that targeting chemokine-GAG interactions may be a promising approach to inhibit chemokine activity (68).

The most prominently studied function of the chemokine network is the cell migration of leukocytes. Yet, cell movements such as haptotaxis, chemokinesis, cell adhesion, and chemorepulsion also fall under chemokine control (63). As leukocyte migration is a critical component of the immune response, a breakdown of chemokine-directed cell migration results in the failure of immune-surveillance and results in a faulty immune response. Chemokine-directed leukocyte migration plays an important role in a number of diseases. Yet, to date, only three clinical therapeutic agents targeting chemokines or chemokine receptors have been approved; Maraviroc^®^ for preventing HIV infection, Mozobil^®^ for hematopoietic stem cell mobilization, and Poteligeo^®^ for patients with relapsed or refractory mycosis fungoides or Sézary syndrome (27).

### 4.2 Chemokine Receptors and Multiple Myeloma

MM cell lines express high levels of the chemokine receptors CXCR3, CXCR4, CCR1, CCR5, and CCR6 (69). CCL3 can interact with CCR1, CCR5, or CCR9 with CCR1 and CCR5 being expressed by human BMSCs and OC precursors. Levels of CCL3 and CCL14 positively correlate with the percentage of bone marrow infiltrating macrophages with *in vitro* and *in vivo* models also suggesting that CCL3, CCL14, and CCL2 may promote chemotaxis of monocytes into the bone marrow (70). The CXCR4/CXCL12 axis has been shown to promote transendothelial migration of MM cells to the endothelium, and CXCR3 interactions contributes to the metastasis of MM (69). CCL2 can be a potent chemoattractant for endothelial cells, basophils, eosinophils, monocytes, and a subset of T-lymphocytes and binds to CCR2 on peripheral blood monocytes, as well as activated B- and T-cells. The upregulation of IL-6 and TNF-α in the MM microenvironment can serve to upregulate the production of CCL2, which is not typically present in normal BME, thereby enhancing the migration of MM cells (69).

NK cells are innate lymphoid cells that play an active role in immunosurveillance against tumors through their secretion of various cytokines and chemokines. Chemokine receptors are located on NK cells and are responsible for the mobilization and extravasation of NK cells as part of the anti-tumor response. There is a downregulation of CXCR3 on NK cells, along with an upregulation of CXCL9, CXCL10 and downregulation of CXCL12, along with a general dysregulation of the CXCR4/CXCL12 axis in the MM microenvironment. Typically, CXCR3 functions to mobilize NK cells while CXCR4 functions in NK cell retention. Thus, the upregulation of CXCR3 and downregulation of CXCL12 serve as exit signals, driving NK cells out of the bone marrow, resulting in an impaired anti-tumor response (71).

Bone-homing tumor cells tend to overexpress chemokine receptors, CXCR4, CXCR6, and CXCR2 that subsequently contribute to the movement of the tumor cells from the bone marrow to other organs by seeking CXCL12, CXCL-16, and CXCL-10 respectively (47). CXCL12 (aka SDF-1), is highly expressed by BMSCs and critical for the homing of MM plasma cells from the peripheral circulation to the bone marrow. However, much remains to be elucidated about the factors that influence MM plasma cells to stray from the bone marrow. An emerging theory is that of bone marrow hypoxia, in which hypoxic MM plasma cells are preferentially mobilized to the peripheral blood due to decreased adhesion and a reduced chemotactic response to BMSCs (72). Hypoxia in the bone marrow induces expression of HIF-1α and HIF-2α, with HIF-2α playing a critical role in MM *via* the expression of CXCL12 on BMSCs. CXCL12 is highly expressed on MM plasma cells and is able to drive osteolysis and angiogenesis in MM patients. HIF-1α is able to upregulate CCR1, the receptor for CCL3, and CCL3/CCR1 signaling can lead to the dysregulation of CXCR4 (72). It has been suggested that the upregulation of CCR1 together with inactivation of CXCR4, drives MM cells from the bone marrow (72). One mechanism might be through epithelial-mesenchymal transition (EMT). This process enables dissociation of cells from the primary tumor mass, invasion through the extracellular matrix, intravasation into blood vessels and colonization of distant organs. Cells that revert to the epithelial state *via* the mesenchymal-epithelial transition are thought to be responsible for metastases (73). Several of the cytokines and chemokines upregulated in MM BME including IGF-1, IL-1β, IL-6 and CXCR7 promote EMT.

The importance of the chemokine network has prompted the exploration of developing molecules to modify the function of chemokines and/or the chemokine receptors. For instance, the CCR5 antagonist Maraviroc (UK-427,857, Selzentry^®^ in the US, Celsentri^®^ elsewhere) by Pfizer is an antiviral agent used in HIV to block viral entry into macrophages. The CXCR4 antagonist Plerixafor (Mozobil^®^, AMD3100) developed by AnorMED and marketed by Genzyme, blocks homing of hematopoietic stem cells to the bone marrow allowing for their collection from the bloodstream as part of the transplantation process (67). Plerixafor, in combination with bortezomib, was evaluated in MM patients and reported an overall response rate of 48.5%, a clinical benefit rate of 60.6%, and a median disease-free survival of 12.6 months, indicating that targeting the BME and its interaction with the tumor environment can help overcome therapy resistance (74). The CCR4 antagonist Mogamulizumab (KW-0761, AMG761, Poteligeo^®^) was approved by the US FDA for the treatment of two major types of cutaneous T-cell lymphomas, showing improved progression-free survival, improved overall survival rates, and reasonable adverse events (75).

While there are hundreds of experimental chemokine receptor antagonists targeting chemokine receptors, currently there are only 2 therapies targeting chemokine receptors in Phase III clinical trials: Leronlimab (PRO 140), a mAb that recognizes CCR5 for COVID-19 pneumonia (NCT04347239) and Gallium-68 labeled Pentixafor for imaging the CXCR4 chemokine receptor (NCT04859959). As treatment with single chemokine antagonist may not be enough to suppress chemotaxis, recent research has focused on dual antagonists such as those targeting CCR2/CCR5 (cenicriviroc, BMS-813160, CNTX-6970, PF-04634817), CXCR1/CXCR2 (DF2156A), and CXCR4/CCR5 (vicriviroc); and several of these agents are in Phase II trials at this time (75). There also exists an idea termed “biased antagonism” in which compounds can selectively inhibit different downstream signaling pathways (Gi versus Gq for example) (76). Identifying biased antagonists of chemokine receptors could provide a unique method for targeting MM as it might allow for an agent to reduce myeloma cell metastasis while leaving neutrophil chemotaxis intact. As CCR1 is a receptor shown to display signaling bias the idea warrants further investigation.

## 5 CCR1 in Multiple Myeloma

### 5.1 CCR1 Signaling

CCR1 was the first CC chemokine receptor to be discovered. Human CCR1 (hCCR1 or CD191) and the mouse counterpart share ~80% amino acid identity with both human and mouse CCR1 binding to CCL3 with high affinity. CCR1 is a highly promiscuous receptor, binding to a plethora of chemokines including CCL3, CCL4, CCL5, CCL6, CCL7, CCL8, CCL9, CCL13, CCL14, CCL15, CCL16, and CCL23 (77). CCR1 is activated through multi-site binding subsequently initiating a cascade of intracellular events that can result in cell proliferation, differentiation, apoptosis, phagocytosis, and/or chemotaxis. Downstream signaling is dependent upon the Gα isoform that is activated with Gα_q_ stimulating phospholipase C and a Ca^2+^ flux, Gα_i_ inhibiting adenylate cyclase, and Gα_12/13_ activating RhoGTPase nucleotide exchange factors (RhoGEFs). Other non-G-protein signaling partners such as β-arrestin are also responsible for downstream events that occur after CCR1 activation following chemokine binding. As different chemokines can activate CCR1 to induce a variety of downstream responses, it appears biased agonism plays a role in signaling adding to the complexity and promiscuity of CCR1 (77).

Given that chemokine receptors are highly expressed on tumor cells and may be responsible for metastases, there has been a long-standing interest in identifying antagonists as therapeutic agents. In particular, CCR1 has been shown to be involved in metastasis for numerous cancers including ovarian, breast, prostate, hepatocellular carcinoma, oral squamous cell carcinoma, non-small cell lung, and multiple myeloma (78). Recent studies using *in vivo* targeted silencing of CCR1 found activation of this pathway was necessary for the differentiation of myeloid-derived suppressor cells and protumoral macrophages (79). However, significant differences in expression and function of CCR1 are present between human and animal models. For example, mouse models show CCR1 as a chemotactic factor for neutrophils but not monocytes. These species differences complicate the translation of CCR1 antagonist research from animal to humans (80) and many clinical studies have proven inconclusive or failed. Even with these challenges, CCR1 antagonists warrant further studies for specific cancers including multiple myeloma.

### 5.2 CCR1 in Multiple Myeloma

CCR1 has a clear role in the BME with effects on the differentiation and functions of OBs and OCs supported by studying CCR1 deficient mice which presented with fewer and thinner trabecular bones and a lower mineral bone density in cancellous bones (81). Cell types in the bone marrow that express CCR1 includes BMSCs, OC precursors, OBs, endothelial cells, and CD34^+^ cells. Many studies have indicated there is a role for CCR1 in MM patients. Multiple human and murine myeloma cell lines such as U266, ARH-77, RPMI-8226, OPM2, MM1S, 5T2 MM, and 5T33 MM express CCR1 (82). *In vitro* models support CCR1’s role in OC formation, function and recruitment in both normal and diseased conditions (37).

CCL3/CCR1 interactions are critical for OC differentiation. CCL3 may influence bone formation by inhibiting OB function through impaired matrix mineralization and suppression of osteocalcin production (77). MM plasma cells secrete high levels of CCL3 (between 30 pg/ml and 200 ng/ml). CCL3 may also originate from BMSCs, OB precursor cells, and both immature and mature OCs (82). CCL3 expression by MM plasma cells is enhanced by their adhesion to BMSCs *via* VLA-4 as well as by inflammatory cytokines. CCL3 enhances MM plasma cell proliferation both directly and indirectly. For instance, CCL3 can stimulate MM cell growth indirectly by triggering the secretion of IL-6 from BMSCs. CCL3 levels are elevated in the bone marrow of most patients with active myeloma (83) and the levels of CCL3 correlate with the extent of bone disease (84). CCL3 levels may be indicative of MM status as higher CCL3 mRNA and protein levels are seen in MM patients expressing the κ light chain subtype versus healthy controls. Additionally, increased levels of CCL3 correlate with increased levels of β2-microglobulin and LH, again, markers of MM disease stage and progression. It has also been shown that CCL3 levels are significantly decreased in MGUS compared to Stage I or Stage II MM patients, suggesting that the chemokine plays a role in disease progression (82).

A role for the receptor for CCL3 (CCR1) in MM is also emerging. CCR1 enhances adhesive interactions between MMs and BMSCs, suggesting it may be important in determining the extent of bone disease. As with CCL3, overexpression of CCR1 was associated with increased disease activity (85). CCR1 antagonists block MM cell migration to CCL3 *in vitro*. Furthermore, antisense, and neutralizing antibodies studies showed downregulation of CCR1 signaling altered disease progression in mouse models of MM (30, 86). Using an *in vivo* mouse model (5TGM1) Dairaghi et al. showed that a CCR1 antagonist could block the formation of mature osteoclasts and reduce osteolytic bone damage with a 90% reduction in tumor burden (31). As a result, some researchers have suggested that CCR1 antagonists may be able to downregulate the effects of CCL3 in MM, decreasing the extent of bone disease, extending remission times, and improving patient survival rates (77).

### 5.3 CCR1 Antagonists

There are a number of issues that make developing small molecule chemokine receptor antagonists difficult including the network complexity, redundancy, promiscuity, biased signaling, and interspecies differences. In addition, there are pharmacological concerns such as the need for near-full receptor occupancy (≥ 90%), and prolonged receptor occupancy that make CCR1 particularly challenging (77). However, the attractiveness of a CCR1 antagonist for MM and potentially other cancers and/or bone complications, cannot be ignored. Further support for the development of CCR1 antagonists comes from clinical trials which have shown the agents to exhibit low toxicity (80). A search of literature and patents revealed the following CCR1 antagonists as previously evaluated or actively in evaluation; AZD-4818, BI-638683, BL-5923, BX-471, C-6448, C-4462, CCX9588, CCX354, CCX721, CP-481715, MLN-3701, MLN-3897, PS-031291/PS-375179, and UCB-35625 (**Table 2**). Each of these compounds is briefly discussed below.

**Table 2:** Literature and patent search results of CCR1 antagonists previously evaluated or actively undergoing clinical evaluation.

CCR1 Antagonist	Structure	Results	References
*AZD-4818*	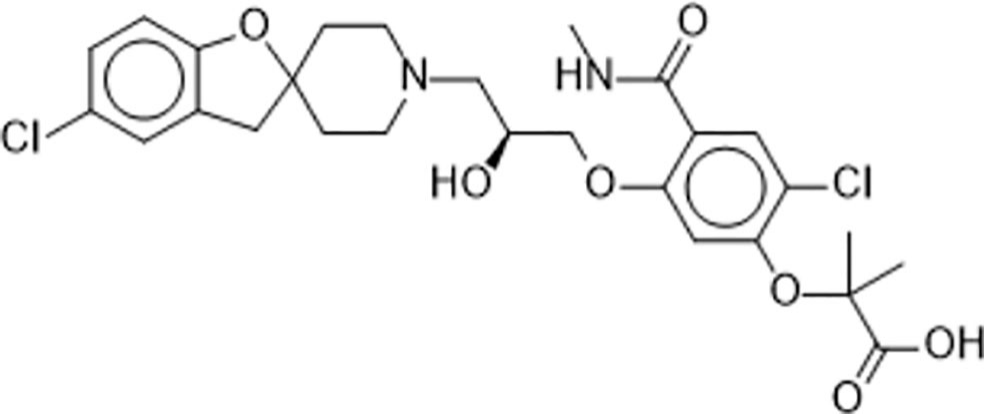	Well tolerated in human model for COPD. *In vitro* and *in vivo* models showed reduction of leukocyte infiltration, inhibition of neutrophil influx in the lungs of rat model, and inhibition of CCL3 chemotaxis of human monocytes.	(87)
*BI-638683*	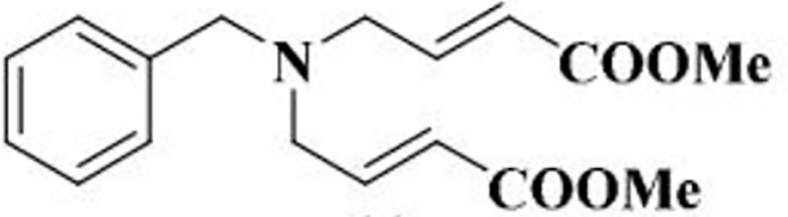	Recruitment status complete however no results posted (Phase I).	(88, 89)
*BL5923*	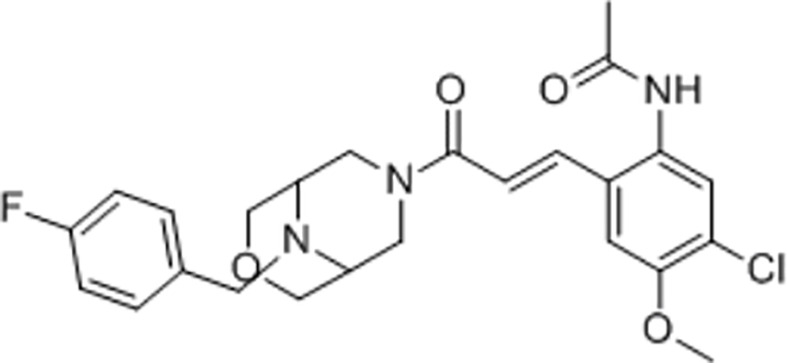	Protection of mouse models from lupus nephritis, diabetic neuropathy, and metastasis of colon cancer.	(90, 91)
*BMS-817399*	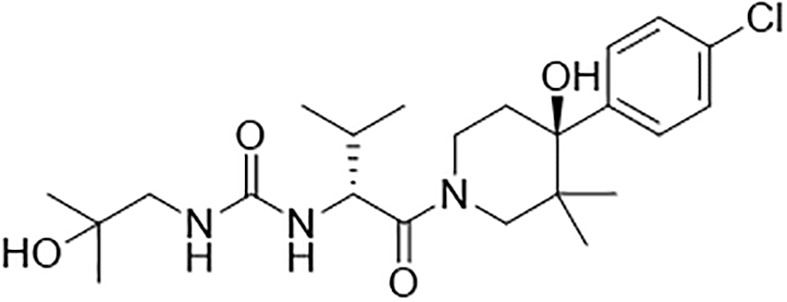	A Phase I trial for BMS-817399 was initiated in 2009 followed by a 12-week, Phase II, multicenter, randomized, double-blind, placebo-controlled study to assess BMS-817399 in patients with rheumatoid arthritis.	(92, 93)
*BX-471*	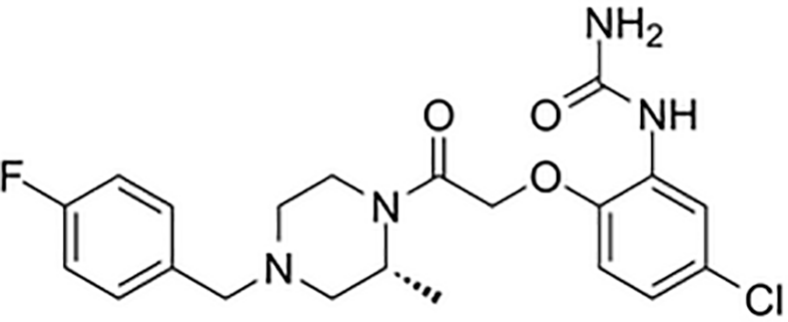	Suppression of CCL5-induced malignant phenotypes and cellular signaling caused by dermatan sulfate epimerase silencing in HCC animal models. Reduction of migration, invasion, and pulmonary metastasis induced by OPN *in vivo* and *in vitro.* BX471 rescued erythropoiesis by hematopoietic stem and progenitor cells.	(94–97)
*C-6448/C-4462*	Structures not disclosed	In 2004 C-6448 entered Phase II trials for multiple sclerosis while C-4462 entered Phase II trials for rheumatoid arthritis. No reports since.	(98)
*CCX9588/CCX721/CCX354*	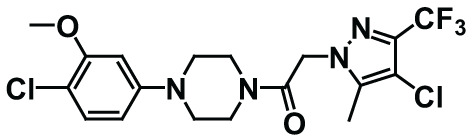	Phase 2 trial with CCX354 showed clinical benefit in RA patients. CCX721 reduced tumor growth in murine 5TGM1 MM model. CCX9588 reduced OPM2 or RPMI-8226 dissemination in intratibial xenograft models of MM. Co-administration of CCX9588 with an anti-PDL1 antibody reduces tumor burden in a breast cancer mouse model.	(31, 33, 99, 100)
*CP-481715*	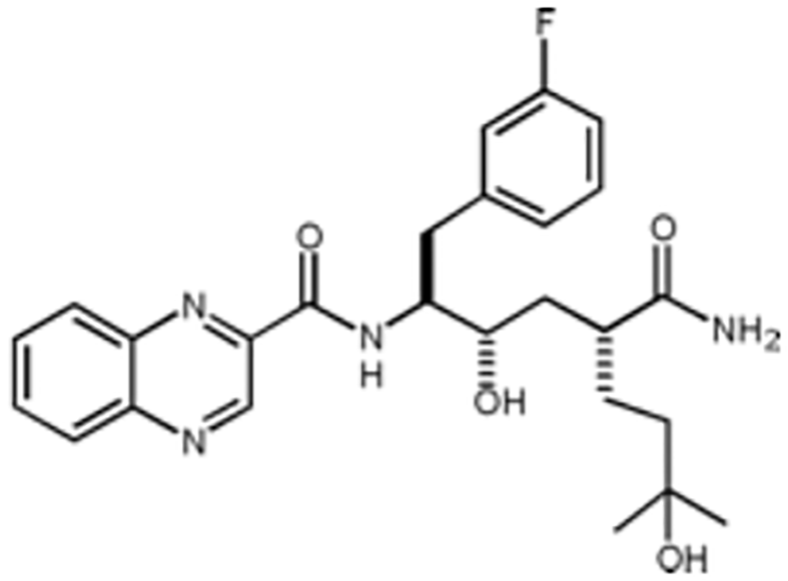	Inhibition of footpad swelling in mouse model. Partial inhibition of allergic contact dermatitis clinical manifestations of erythema and nickel reactions.	(101, 102)
*MLN-3701*	Structure not disclosed	Single oral doses up to 100mg as generally safe and well-tolerated in human clinical trial (Phase I).	
*MLN-3897*	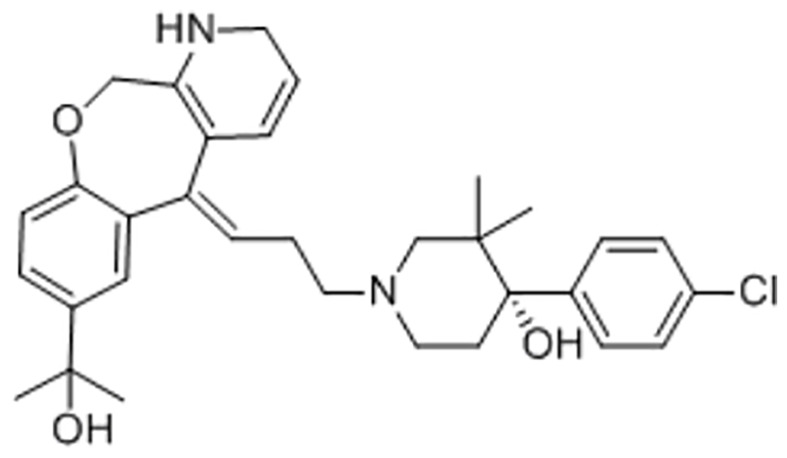	Impairment of OC formation and function, inhibition of osteoclastogenesis and OC activity, reduced CCL-3 induced cell migration, inhibition of protective effects of OCs in MM.	(32, 103)
*PS-031291/PS-375179*	Structures not disclosed	Program discontinued for multiple sclerosis and rheumatoid arthritis and suspended for cancer in preclinical stage.	(104, 105)
*UCB-35625/J113863*	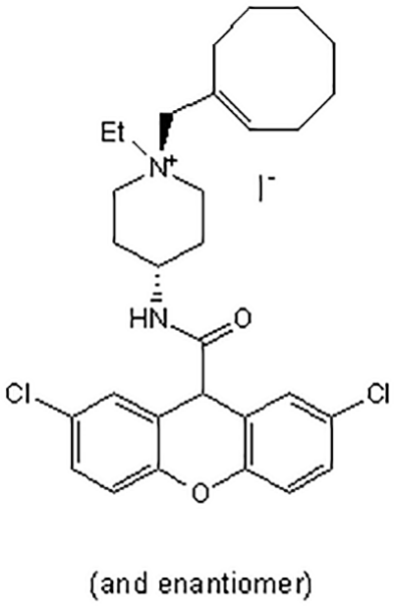	Improved paw inflammation and joint damage, and decreased cell infiltration into joint space of mouse models with collagen-induced arthritis Blockage to HIV viral entry with CCR3 using HIV strains.	(106–108)

#### 5.3.1 AZD-4818

Inhaled AZD-4818 was evaluated in a double-blind, placebo-controlled study (NCT00629239), in patients afflicted with COPD in which AZD-4818 at 300 μg twice daily *via* Turbuhaler^®^ was well-tolerated. *In vitro* models, AZD-4818 inhibited the binding of CCL3 to CCR1 in human, rat, mouse, and dog and the inhibition of CCL3 chemotaxis of human monocytes. *In vivo* models showed a reduction of leukocyte infiltration and inhibition of neutrophil influx in the lungs of rat models (87). However, it was concluded that AZD-4818 showed no beneficial effects in COPD as a monotherapy and could perhaps require a drug combination and/or a non-inhalation method for best results.

#### 5.3.2 BI-638683

Results for the evaluation of BI-638683 were provided in a poster at EULAR 2014 (88). BI-638683 is reported in a Phase I clinical trial (NCT01195688) in the U.S. for safety and tolerability in healthy male subjects as of June 2011, with recruitment status showing complete but no results posted (89). There is also a reference to a Phase I clinical trial in Germany for the treatment of rheumatoid arthritis with no further developments reported as of March 2018.

#### 5.3.3 *BL5923*

BL5923 is a selective CCR1-selective small-molecule antagonist that lacks any significant binding to human CCR2, CCR4, CCR5, CCR6, CCR7, CXCR1, CXCR2, OR CXCR3. BL5923 has been shown to protect mice from lupus nephritis, diabetic nephropathy, and metastasis of colon cancer to the liver. A genetic deficiency in mice for CCR1 showed diminished renal tissue injury and improved survival in a mouse model infected with candidiasis. A follow-up study of a pharmacological blockade of CCR1 with BL5923 in immunocompetent mice infected with systemic candidiasis *in vivo* resulted in significant improved survival rates 2-weeks post-infection (50%), longer median survival time (10 days vs 3 days), decreased tissue fungal burden in the kidney (p = 0.0185), and less extensive renal tissue invasion when compared to the control mice (90). BL-5923 is able to block immature myeloid cell accumulation, metastatic colonization, and significantly extend the survival times of tumor-bearing mice with colon cancer (91). BL5923 was administered to mice injected with mouse (CMT93) or human (HT29) colon cancer cells which displayed a significantly improved mean survival from 37 days post-injection to 62 days post infection (CMT93) and 84 days post-injection to 113 days post injection (HT29). For mice injected with luciferase-expressing CMT93 cells, BL5923 significantly reduced the luminescence level by day 14.

#### 5.3.4 BMS-817399

Information on BMS-817399 was reported (92) following disappointing results from a Phase II 12-week proof of concept study (NCT01404585) indicating the drug was safe and well-tolerated but showed no evidence of clinical efficacy in rheumatoid arthritis patients with moderate to severe disease activity with inadequate response to methotrexate (93).

#### 5.3.5 BX-471

BX-471 was identified by Berlex and initially tested in Phase I studies for multiple sclerosis. However development was stopped after the Phase II study failed to demonstrate a positive clinical end point (reduction in the number of new inflammatory CNS lesions) (94). CCL5 activity is mediated through its binding to CCR5, CCR3, and CCR1 but only CCR1 is expressed in hepatocellular carcinoma cells (HCC). As CCR1 is overexpressed in HCC, and CCL5 is thought to be responsible for the abnormal activity of dermatan sulfate epimerase in HCC, BX-471 was used to analyze the role of CCL5/CCR1. The investigators concluded that BX-471 was able to suppress CCL5-induced malignant phenotypes and cellular signaling caused by dermatan sulfate epimerase silencing in HCC cells (95). Further studies using a knockdown of CCR1 with BX-471 indicated a reduction of migration, invasion, and pulmonary metastasis induced by osteopontin (OPN) *in vitro* and *in vivo* (96). As patients with high levels of OPN and CCR1 are associated with a poor prognosis, CCR1 antagonists may have the potential to reduce metastasis in HCC patients. Recently, BX471 was used in the co-culture system of CD34^+^ cells and bone marrow plasma cells from MM patients to rescue erythropoiesis (97).

#### 5.3.6 C-6448/C-4462

Two xanthene carboxamide derivatives (98) were taken into Phase II clinical trials by Merck (C-6448 for multiple sclerosis and C-4462 for rheumatoid arthritis) in 2004 but neither program was continued.

#### 5.3.7 CCX9588, CCX721, CCX354

The first-generation CCR1 antagonist CCX354 was shown to have clinical benefit in RA patients in a Phase II trial (NCT01242917) (99). Pre-clinical studies with an analog of CCX354, CCX721 found that it reduced tumor growth and osteolysis targeting OC and their precursors in the murine 5TGM1 MM model (31). A second-generation CCR1 antagonist, CCX9588, was reported to block CCR1-mediated chemotaxis with an IC_50_ of 0.1 nM with THP-1 cells (109). CCX9588 was shown to significantly reduce OPM2 or RPMI-8226 dissemination in intratibial xenograft models of MM (33). Co-administration of CCX9588 with an anti-PDL1 antibody was also shown to reduce tumor burden in a breast cancer mouse model (100).

#### 5.3.8 CP-481715

CP-481715 is a CCR1-selective antagonist specific for hCCR1 with limited use in mouse models. CP-481715 was shown to inhibit footpad swelling and decrease the amount of IFN-γ and IL-2 produced by isolated spleen cells taken from the hCCR1 knock-in mice (101). A study of CP-481715 in allergic contact dermatitis patients (NCT00141180) revealed a partial inhibition of clinical manifestations with the reduction of nickel reactions (p = 0.01), and a reduction in erythema (p = 0.06) (102). It is believed that in the case of allergic contact dermatitis, more than one chemokine should be targeted as multiple members of the chemokine network are involved, and cannot be sufficiently modulated *via* CCR1 monotherapy.

#### 5.3.9 *MLN-3701/*MLN-3897

MLN-3701 is referenced in a clinical trial in Japan (C11001) as a Phase I, randomized, double-blind, placebo-controlled, sequential single ascending oral dose study to assess the safety, tolerability, pharmacokinetics, and F365 pharmacodynamics, in healthy male subjects, completed in March 2006. Findings state single oral doses of MLN-3701 up to 1000mg as generally safe and well tolerated.

MLN-3897 demonstrated significant impairment of OC formation (40%) and function (70%) in the tumor microenvironment of MM. MLN-3897 is able to inhibit osteoclastogenesis and OC activity by downregulating c-fos signaling which impairs multinucleation, Akt inhibition which reduces CLL3-induced MM cell migration, and the inhibition of the protective effects of OCs in MM survival (32). In a study evaluating MLN-3897 in combination with methotrexate in patients with rheumatoid arthritis, results showed MLN-3897 was well-tolerated, and showed no signs of systemic immunosuppression. However, a dose of 10mg once daily, did not show a significant difference when compared to the “per-protocol” population (103).

#### 5.3.10 PS-031291/PS-375179

PS-031291 replaced PS-375179 as the lead CCR1 receptor antagonist from Pharmacopeia. However, work on PS-031291 was discontinued during preclinical development shortly after acquisition of the company by Ligand. The structures are likely similar to those published by Merritt et al. (104, 105).

#### 5.3.11 UCB-35625/*J113863*

J-113863 and its enantiomer UCB-35625 are high affinity dual CCR1/CCR3 antagonists (106). A mouse model of collagen-induced arthritis treated with J-113863 improved paw inflammation and joint damage, and decreased cell infiltration into the joint space (107). Inhibitory effects of UCB-35625 at CCR1 and CCR3 are potent and specific as proven by biological assays of cellular activation. As CCR1 and CCR3 are major eosinophil chemokine receptors, UCB-35625 displays promise in treating allergic inflammatory diseases and as a blockage to HIV viral entry for CCR3 using HIV strains (108).

Some CCR1 antagonists are orthosteric in nature (CP481,715) while others are allosteric (BX-471). Allosteric antagonists may be probe-dependent (ligand bias), pathway-dependent (functional selectivity), and cell type dependent (context dependent). To address ligand bias, functional selectivity, and context-dependent signaling, studies should be performed using physiologically relevant systems and appropriate end points. Unfortunately, biological information available for many of the mentioned CCR1 antagonists is quite limited often being restricted to what was provided in patent applications (110).

## 6 Discussion

The depth of response with MM therapy correlates with long-term outcomes such that patients with a complete response have longer progression-free survival and overall survival. Identifying high risk patient-specific factors has led to a greater understanding of the disease, and modification of diagnostic and prognostic procedures has increased survival, but MM remains an incurable disease. Current pharmacologic treatments vary in their mechanisms of action, but most require multiple drug combinations, and may elicit intolerable side effects and only minimally extend the remission time for MM patients. The chemokine network represents a novel target for MM. Chemokines and their receptor can influence the immune system dysfunction often present in MM to return the balance to a more appropriate immune response. Chemokine receptor antagonists have proven beneficial for conditions such as HIV, and hematopoietic stem cell transplantation but have not been largely exploited as therapeutic agents. Animal models suggest that CCR1 plays an important role in a myriad of MM disease symptoms including reduced OB differentiation, increased OC activity, and metastasis. It may be possible for a CCR1 antagonist delivered as a monotherapy or in combination with other drugs to potentially minimize or eliminate such effects.

Many CCR1 antagonists have failed or provided minimal results during clinical trials for a wide variety of diseases from multiple sclerosis and rheumatoid arthritis to neuropathic pain and allergic contact dermatitis. This may be due to the broad approach of these studies. For example, it was hypothesized that inhalation of AZD-4818 may not be the correct way to deliver the medication for the asthma trial and CP-481715 may not sufficiently reduce allergic contact dermatitis because of the numerous chemokines present in the skin. Studies with CCR1 antagonists may need to follow a stricter approach with more stringent control of their study design to gain meaningful insights. Future *in vitro* studies should analyze the efficacy of various CCR1 antagonists, comparing old versus new compounds, looking not only for the inhibition of chemotaxis but additional activity (osteoclastogenesis, expression of adhesion molecules). For MM, study designs should specify the Stage of MM, and the best formulation and dosing of the compound. There is much to learn from the chemokine network and its roles in both the healthy and diseased states, but it is certain that this network provides a prime opportunity for advances in medical treatment.

## Author Contributions

SE and AG drafted the manuscript. Both authors contributed to the article and approved the submitted version.

## Funding

This work was supported by a Midwestern University College of Pharmacy Downers Grove Faculty Research Grant.

## Conflict of Interest

The authors declare that the research was conducted in the absence of any commercial or financial relationships that could be construed as a potential conflict of interest.

## Publisher’s Note

All claims expressed in this article are solely those of the authors and do not necessarily represent those of their affiliated organizations, or those of the publisher, the editors and the reviewers. Any product that may be evaluated in this article, or claim that may be made by its manufacturer, is not guaranteed or endorsed by the publisher.
